# Investigation of an integrated standardised pain assessment and management tool in addition to usual care versus usual care alone in oncology outpatient clinics for adults with pain: the CAPTURE cluster randomised pilot trial protocol. ISRCRN86926298

**DOI:** 10.1186/s40814-025-01714-5

**Published:** 2025-10-30

**Authors:** Olivia C. Robinson, Elaine G. Boland, Florence Day, Marie Fallon, Amanda Farrin, Kate Flemming, Sean Girvan, Sue M. Hartup, Adam Hurlow, David Meads, Catriona R. Mayland, John L. O’Dwyer, Simon Pini, Suzanne H. Richards, Daniel Swinson, Michelle Collinson, Matthew R. Mulvey

**Affiliations:** 1 Leeds Institute of Health Sciences, University of Leeds, Worsley Building, Clarendon Way, Leeds, LS2 9NL UK; 2 Leeds Institute of ClinicalTrials Research, University of Leeds, Worsley Building, Clarendon Way, Leeds, LS2 9NL UK; 3 Palliative Medicine, Hull University Teaching Hospitals NHS Trust, Hull, HU15 5JQ UK; 4 St James’s University Hospital, Leeds Teaching Hospitals NHS Trust, Leeds, LS9 7TF UK; 5 Cancer Research UK Edinburgh Centre, MRC Institute of Genetics & Molecular Medicine, University of Edinburgh, Edinburgh, EH4 2XR UK; 6 School of Medicine and Population Health, University of Sheffield, Sheffield, S10 2SJ UK; 7 Sheffield Teaching Hospitals NHS Foundation Trust, Sheffield, S10 2SJ UK; 8 Department of Health Sciences, The University of York, York, YO10 5DD UK

**Keywords:** Cancer, Pain, Cluster trial, Pilot, Outpatient setting

## Abstract

**Background:**

Each year in the UK approximately 367,000 people are diagnosed with cancer of whom half will experience moderate to severe chronic pain and a third are undertreated for their pain. Most people with cancer are cared for at oncology outpatient services where there are no standardised approaches for managing pain. As a result, cancer patients are at risk of receiving inadequate care for pain. There is a need for a standardised approach to pain management within oncology outpatient services.

**Methods/design:**

The aim of this pilot trial is to establish the feasibility of conducting a multi-centre clustered-randomised trial of an integrated standardised pain assessment and management programme integrated within routine care at oncology outpatient services in the United Kingdom National Health Service (NHS).

We will conduct a two-arm pilot cluster randomised trial with nested process evaluation to evaluate the feasibility and acceptability of trial processes, establish fidelity of intervention implementation, estimate variability in outcomes and feasibility of future economic evaluation. Twelve outpatient services (clusters) from at least two NHS tertiary oncology referral centres (sites), in the North of England will be randomised (1:1) to deliver a pain management programme plus usual care or usual care alone and will recruit a total sample of 180 participants. Adults attending a participating outpatient service who self-report a score of ≥ 3 on the 0–10 Numerical Rating Scale (NRS) for worst pain in the past 72 h in any part of their body, and will be available for 1-week follow-up will be eligible. Participant self-reported questionnaires will be collected at baseline, 1-week, 1-month, and 2-months with medical record review at 1-month and 2-months. Progression to a future trial will be based on pre-defined criteria associated with eligibility and consent rates, follow-up and intervention delivery and acceptability.

**Discussion:**

Little research has described optimal ways to implement a standardised pain assessment and management programme into oncology outpatient services. The strengths of the pilot trial are its sample size, number of clusters, and planned evaluation of trial processes and intervention fidelity to provide robust trial evidence to fully inform a future definitive phase III multi-centre cluster randomised trial within the UK NHS.

**Trial registration:**

The CAPTURE pilot trial is registered on the ISRCTN registry (86,926,298).

## Background

Each year in the UK approximately 367,000 people are diagnosed with cancer. The prevalence of cancer-related chronic pain is estimated to be more than 70% [1]. Approximately 50% of all patients with cancer will experience moderate to severe cancer-related chronic pain (i.e. pain related to cancer and its treatment); and a third of patients with cancer are undertreated for their cancer-pain [2, 3]. Under-treatment of cancer pain reduces patients’ quality of life, [3] and increases healthcare service use and costs [4]. For patients, the burden of chronic cancer-related pain is associated with anxiety and depression, [5] significant reduction in physical and emotional wellbeing, as well as overall reduction in quality of life [6]. Clinicians report that a lack of awareness of pain management guidelines and poor knowledge about pharmacological pain management are the most common barriers to optimal pain management [7, 8]. At a service level, uncontrolled pain is the most common reason for cancer patients contacting GP out of hours services [9].

Numerous guidelines on managing cancer pain have been published in the last 25 years [1, 10]. Trial data indicate that adherence to these guidelines can improve quality of care and pain outcomes for patients with cancer. [11–15] However, in the UK the majority of people with cancer are cared for at oncology outpatient services where there are no standardised approaches for managing pain [10]. As a result, cancer patients receive inadequate and inconsistent care for pain [3, 16, 17]. Systematic review data highlight the need for a standardised approach to the assessment and management of chronic cancer-related pain [11, 18]. As the majority of cancer patients are cared for at oncology outpatient services, there is a clear need for a standardised approach to pain management within these services. This has been highlighted as an NHS health service priority [19, 20].

The Edinburgh Pain Assessment and management Tool (EPAT) was developed for use in a hospital ward setting [12]. EPAT is a simple cancer pain assessment and management tool designed to prompt clinicians to systematically assess and manage cancer pain across the duration of a care episode. Pain scores are used to guide clinical decision making and treatment using linked treatment algorithms. Therefore, EPAT is both a pain assessment and a pain management intervention. In previous implementation, EPAT significantly reduced cancer-related pain in patients on oncology wards and led to more appropriate analgesic prescribing, without higher doses [12].

EPAT consists of four core components: screening, detailed assessment, prescribing, and reassessment. EPAT was designed to be integrated into standard clinical practice by implementing it within existing policies on hospital oncology wards. As such, EPAT works optimally with the contextual factors associated with a hospital ward environment. Prior to the pilot trial, a theoretically informed process of contextual adaptation using the ADAPT guidelines [21] was undertaken to adapt EPAT from its original ward setting for use in oncology outpatient services. This involved a series of qualitative interviews to map existing pain management processes in oncology outpatient services [22]. In a co-design process involving oncology healthcare professionals, each component of EPAT was mapped onto the new setting to identify potential mismatch and subsequent need for modification. This was a key step in the process of adaptation [23]. Each stage of the research was informed by evidence and theory of complex intervention adaption [23–25].

Challenges to implementing optimal pain management procedures into oncology outpatient services remain. Mackhlouf et al. [8] demonstrated that oncology clinicians’ lack of pain management knowledge and fears of opioid addiction prevent effective cancer pain management. Adam et al. [11] demonstrated that basic pain intensity screening (using 0–10 scales) has little impact on pain outcomes for cancer patients or prescribing behaviour of clinicians. This was due, in part, to a lack of guidance for clinicians on how to use pain data. Oldenmenger et al. [13] and Williams et al. [14] both found that tailored pain education for patients attending oncology outpatient services did not lead to improved cancer pain management. A common feature of these two studies is the lack of an explicit implementation strategy to support integration and uptake of routine pain assessment (aimed at clinicians) and self-management information (aimed at patients). Implementing effective pain management processes within routine oncology outpatient services has yet to be successfully achieved and this will be the focus of the CAPTURE pilot trial.

### Overall aim

In the long term, we aim to establish whether a standardised pain management programme integrated within routine care at oncology outpatient services can reduce the impact of chronic pain on individual patients with cancer and the burden on out of hour’s services. To achieve this, we need to establish the feasibility of undertaking a definitive phase III multi-centre cluster randomised trial within the UK National Health Service (NHS).

### Objectives

The objectives of the CAPTURE pilot trial are to:

1. Establish eligibility, recruitment, retention, and follow-up rates to inform the design of a future phase III RCT.
2. Assess the acceptability of the intervention and protocol to healthcare professionals and patients with cancer.
3. Assess the extent to which oncology healthcare professionals can deliver the intervention with competency and fidelity (i.e. deliver the components of the intervention as intended) following brief training.
4. Gather preliminary data on the effect of the intervention via exploration of between-group change in outcomes.
5. Establish the feasibility of an economic evaluation of EPAT and obtain preliminary estimates of cost-effectiveness.

## Methods

### Trial design

We will use a multi-centre, two-arm, pilot cluster randomised controlled trial, with a nested qualitative process evaluation. Twelve outpatient services (clusters) from at least two NHS tertiary oncology referral centres (sites) in the North of England will be randomly allocated (1:1) to deliver EPAT + usual care or usual care alone. 180 eligible patients will be recruited.

### Setting, clusters, and randomisation

Oncology outpatient services will be eligible for inclusion if they:

1. Care for patients with a diagnosis of cancer.
2. Do not currently have a standardised pain assessment and management programme integrated within routine practice.
3. Provide written informed consent to support participation.

**Fig. 1:**
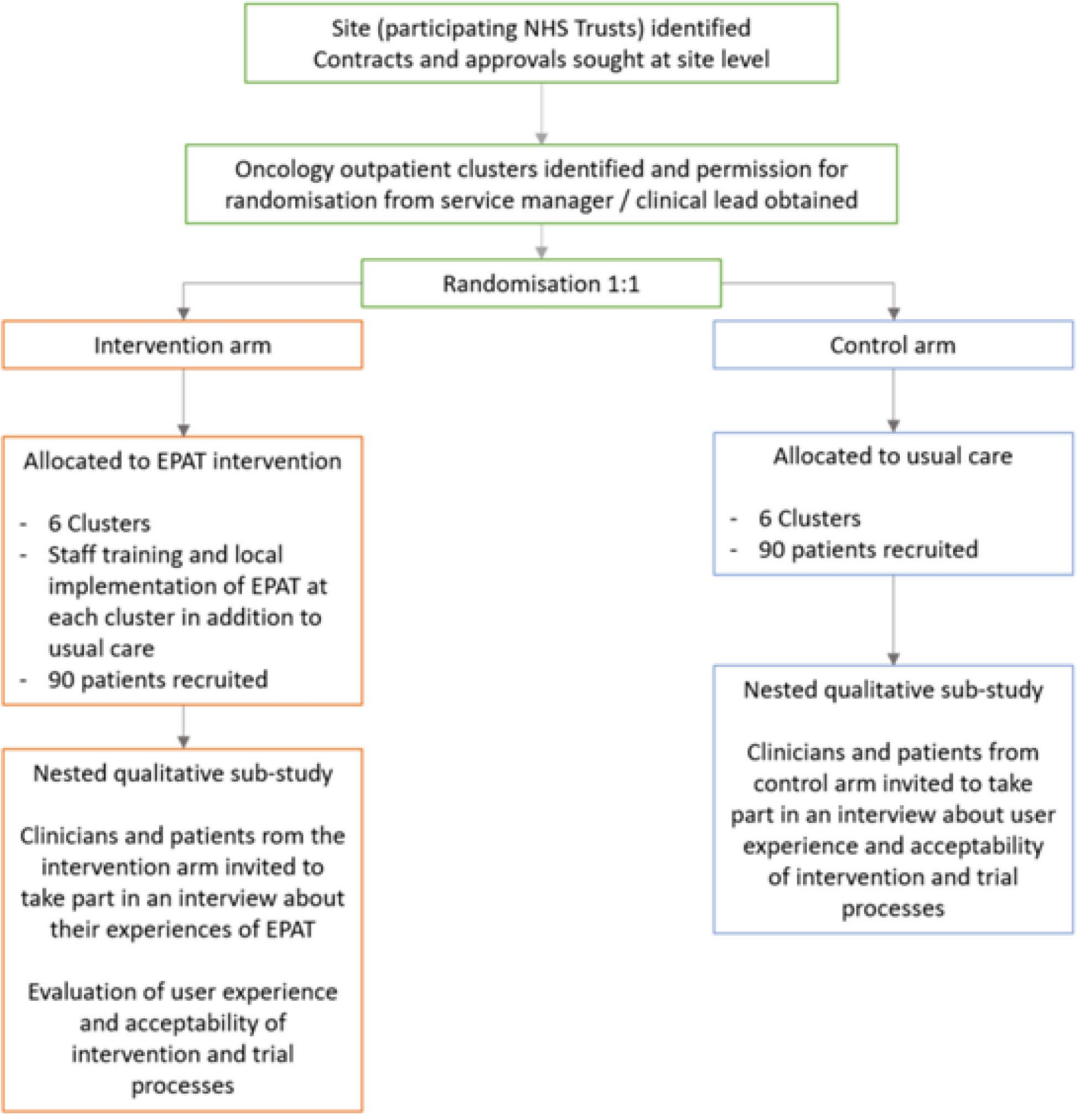
Service-level Randomisation

Each site will be required to have obtained local trust approvals and have undertaken a site initiation meeting within the trial team before services are randomised and participant recruitment starts. Following confirmation of eligibility, services will be randomised (1:1) to deliver EPAT plus usual care or usual care alone (Fig. 1) by the trial statisticians (MC, SG) at the Leeds Clinical Trials Research Unit (CTRU). Minimisation, incorporating a random element will be undertaken by hand to ensure treatment arms are balanced with respect to site, cancer type (i.e., breast vs. lung vs. prostate vs. bowel vs. upper GI vs. haematology vs. bone metastasis) and pain prevalence (high vs. low).

### Methods for protecting against bias

We will recruit and randomise clusters where healthcare professionals (HCPs) have not been involved in previous phases of the research programme [22]. EPAT materials and intervention training will not be available to clusters allocated to usual care alone. We will monitor potential contamination and record details of HCP movement between clusters.

Participants, HCPs, research nurses (RNs), researchers (OR) and CTRU staff will not be blind to the cluster allocation. However, researcher (MM) will be unaware of cluster allocation when supporting telephone follow-up.

### Participant eligibility criteria

Patients with cancer will be recruited from participating oncology outpatient services to provide patient reported outcome data and medical notes review data. Patients will be eligible if they are: attending a participating outpatient service during the trial period; are aged 18 years or over; have a diagnosis of cancer; and self-report a score of ≥ 3 on the 0–10 Numerical Rating Scale (NRS) for worst pain in the past 72 h (including common pain descriptors such as: aching, unpleasant, niggling, discomfort, dull ache, cramp, throb, pinch, sharp, sting) in any part of their body. Patients will be ineligible if they are: deemed by clinical judgement to be too ill to take part (including those with severe mental health problems); considered by their clinical teams to be actively dying; unable to complete a NRS in English; not expected to be available for the first follow-up data collection (1-week). Eligibility waivers to inclusion/exclusion criteria are not permitted.

### Participant identification, approach, and recruitment

Research nurses (i.e., member of the direct care team) embedded within the clinical teams with access to clinic lists in each recruiting cluster will identify potentially eligible patients. Patients will be approached by the research nurse via a telephone call, post, or in-person. Patients approached via telephone, will be sent an invitation pack via email or post. Patients approached by post, will be invited to contact the named research nurse to express an interest in participating in the trial. Patients approached in-person (on the day of their clinic appointment) will be given a verbal description of the study, and an invitation pack to read. It is intended that most patients will be recruited via the telephone approach.

Research nurses will complete a screening log for all patients with cancer who are ≥ 18 years old and attending a participating outpatient service (first three inclusion criteria). Anonymised data on age, sex, ethnicity, year of cancer diagnosis, primary cancer type and tumour type and whether a patient is registered will be collected. Reasons patients were ineligible or declined participation will be recorded.

Informed consent will be obtained by trained research nurses prior to the patient undergoing procedures that are specifically for the purposes of the trial and are out-with standard routine care at the participating site. Patients will be able to provide informed consent over the telephone or in-person. The consent form will be sent to the CTRU via secure electronic transfer. All participants will be asked to supply their email address and telephone number, to allow telephone/SMS reminders to be sent when follow-up questionnaires are due.

**Fig. 2:**
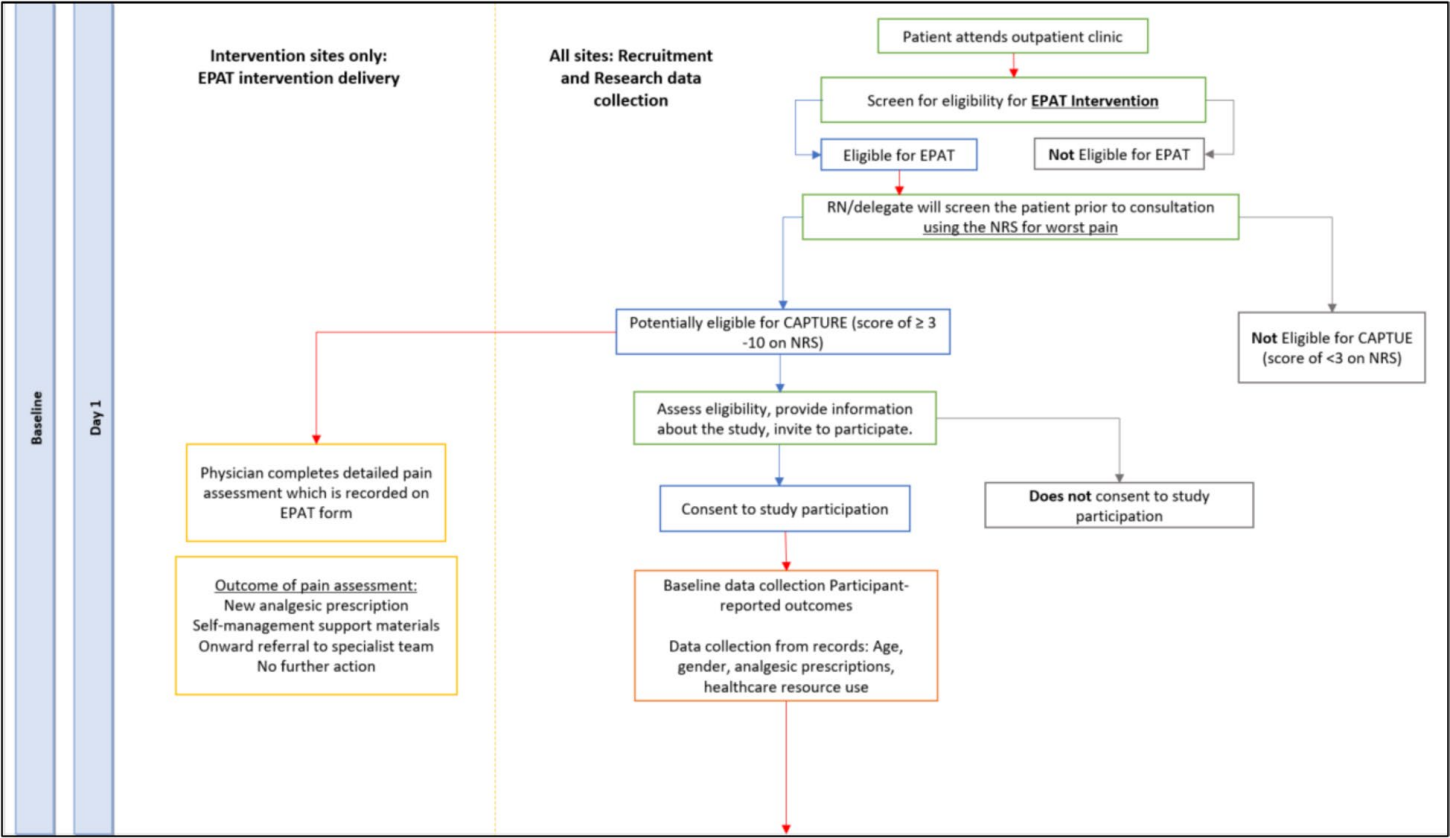
Recruitment and baseline data collection

Once consent and baseline questionnaires have been completed, the research nurse will register the patient to the trial using the CTRU online automated 24-h registration system. Recruitment and baseline data collection procedures are outlined in Fig. 2.

### Description of intervention and usual care

Intervention clusters will receive EPAT plus usual care which will commence following HCP training and run for a maximum of 12-weeks per participant. The EPAT intervention will include: a training module to support clinician education; a pain screening conducted by clinic nurse/clinician (step 1); a detailed pain assessment (step 2) conducted by a physician or clinical nurse specialist for patients with a pain screen ≥ 3/10 on a 0–10 NRS of worst pain in past 72 h; a basic pain management algorithm/protocol to guide analgesic prescribing and/or onward referral to a pain specialist; and, patient resources to support self-management. EPAT will be in paper form, attached to the front of patient’s notes and implemented within existing policy. All patients with cancer (including those not recruited to the trial) attending intervention clusters that have a pain score of ≥ 3/10 will receive the intervention.

At least one HCP from each intervention cluster will take on the role of ‘EPAT champion’ to lead local implementation of the intervention, following training, with support from the trial team. Intervention champions will: have oversight of the relevant outpatient service; have direct interaction with patients and their medical notes; and have direct contact with clinical staff responsible for pain management. Oncologists and oncology nurses working in the cluster will be trained to deliver the intervention by the EPAT champion prior to the start of participant recruitment to ensure the intervention is established as routine practice. Champions will be supported by the research team via weekly catch-up calls and monthly video-call conferences with champions at other services to create a community of practice that will enable knowledge sharing. Data to support exploration of fidelity of intervention delivery will be collected. EPAT will not be available for clusters randomised to deliver usual care alone.

Participants in all clusters, including those randomised to receive EPAT, will receive usual care. It is anticipated that usual care will consist of appropriate individual pain assessment by nursing and medical staff, followed by a management decision. At present in the UK, this part of cancer care is not carried out in a structured, systematic fashion. While pharmacological management is based on the principles of WHO guidelines, the way in which these guidelines are used is not standardised. Pain management provided will be recorded and data describing usual care provision will be collected.

### Outcomes and data collection

The primary outcomes for this trial are the eligibility, recruitment, retention, and follow-up rates. Secondary outcomes include acceptability, intervention fidelity, feasibility of economic evaluation, pain, quality of life, psychological health, and common symptoms.

Baseline data collection will include participant identifiers, demographics (ethnicity, marital status, employment, and education), cancer-specific details (cancer type and stage and date of diagnosis) and pain-specific details (primary cancer site causing pain, clinical diagnosis of pain and analgesic prescribing) (Table 1).

**Table 1:** Description of outcome and process measures, including their timing and method of data collection

Assessment	Measure	Method of completion	Screening	Baseline	1-week	1-month	2-months
**Recruitment processes**							
Screening	Screening form	RN (from clinic screening list)	X				
Contact details	Patient telephone number	RN (from medical records)	X	X			
Eligibility	Researchers will ask the patient if they have had any pain in the past 72-h	RN (from participant)	X	X			
Consent	Consent form	Participant	X	X			
Participant registration	CRF	RN	X	X			
**Pain**							
Pain	BPI	Participant self-report	X	X	X	X	X
**Health & Wellbeing**							
General health-related Quality of life	5Q-5D-5L	Participant Self-report		X	X	X	X
Anxiety	GAD-7	Participant Self-report		X		X	X
Depression	PHQ-8	Participant Self-report		X		X	X
Symptoms	EASA	Participant Self-report		X		X	X
**Participant characteristics**							
Demographics	Basic demographics: age, ethnicity, marital status, sex, highest achieved education level	RN (from participant)		X			
Medical history	Recent medical history, cancer type and stage	RN (from participant)		X			
Medications and referral check plus healthcare resource use	Analgesic prescriptions, specialist service referral, healthcare resource use	RN (from medical records)		X		X	X
Healthcare resource use	specialist service referral, healthcare resource use	Participant self-report		X		X	X
**Other**							
Usual care	Site level usual care offered and accessed	Participant interviewed about use of usual care during process evaluation					X
Intervention training	Evidence of intervention training delivered to clinic staff by intervention champion recorded on CRF	Researcher at University of Leeds		X			
Intervention delivery and fidelity	Completion of EPAT forms by outpatient clinical staff, evidence of EPAT completion recorded on CRF by research nurse for all participants (who consented to provide outcome data) at one-month and two-month follow-up time points	Researcher at University of Leeds				X	X

**Fig. 3:**
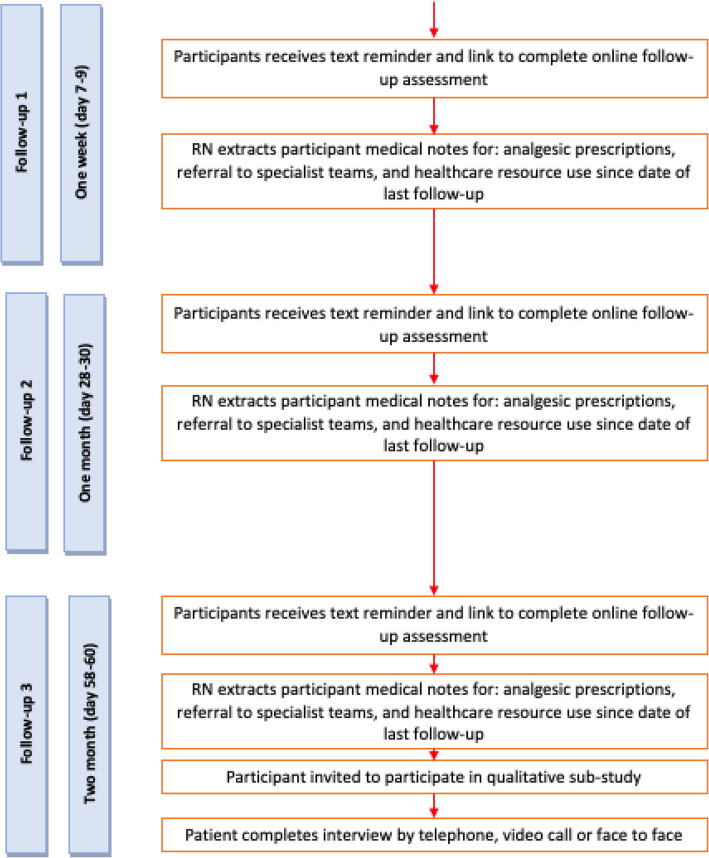
Follow-up data collection

Participants will be asked to complete participant reported outcome measures at baseline, 1 week, and 1- and 2-months post randomisation. At baseline, measures can be completed over the telephone with a research nurse or in-person prior to a participant’s outpatient appointment. Follow-up data will be collected by post or via an online platform (REDCap) supplemented by reminders including telephone, and SMS prompts (Fig. 3).

Participant reported outcome measures include:

*Pain:* The short form Brief Pain Inventory (BPI) [26] is a 9-item tool to assess the severity of pain and its impact on daily function.

*Quality of Life: EQ-5D-5L *[27] is a 5-item generic health related QoL measure.

*Psychological Health:* The Generalised Anxiety Disorder 7-item Scale (GAD-7) [28] is a seven-item measure of anxiety. The Patient Health Questionnaire (PHQ-8) [29] is an eight-item measure of depression.

*Common Symptoms:* The Edmonton Symptom Assessment System (EASA) [30] is a ten-item tool designed to assess common symptoms in palliative care patients.

*Resource Use:* The Health Service Use Questionnaire (HSUQ) is a brief, bespoke questionnaire designed to capture patients’ health services use across 5 key areas: hospital services, out-of-hours services, hospice services, travel to and from services, and support from family and friends.

### Safety

In this population it is anticipated that participants will experience acute illnesses, infection, new medical problems, and deterioration of existing medical problems. This could result in hospitalisation, hospital re-admission, disability, incapacity, or death. Therefore, any events fulfilling the definition of an Adverse Event (AE) or Serious Adverse Event (SAE) will not be reported unless they fulfil the definition of a reportable event or a Related and Unexpected Serious Adverse Event (RUSAE).

SAEs and RUSAEs will be monitored and assessed through case report forms (CRFs), contact with the research team/principal investigator at site, research team at the University of Leeds and via follow-up questionnaires. If either research team becomes aware that a trial participant that has died, a CRF will be completed to capture the event. As this is expected within this population, it will not be subject to expedited reporting to the main Research Ethics Committee (REC).

Reportable events include RUSAEs, hospitalisations that have been associated with uncontrolled pain (inpatient stays and A&E attendances), contacting out of hours services and Clinical Nurse Specialist (CNS) for uncontrolled pain. Hospitalisations (except for those associated with uncontrolled pain) are expected within this population and will not be subject to expedited reporting.

### Sample size

A formal power calculation is not required for this pilot trial, as it is not designed to estimate effectiveness. However, the sample size must be sufficient to establish eligibility, consent, recruitment, and dropout rates and assess the acceptability and fidelity of EPAT to inform a future trial. Teare et al. [31] recommends a minimum of 60 participants per arm for a pilot trial with a binary primary outcome variable. Allowing for 30% loss to follow-up and rounding to allow balanced recruitment across clusters, a total sample size of 180 participants (90 participants per arm; 15 patients per cluster) will be sufficient [32, 33].

### Analysis

A detailed statistical analysis plan will be written and signed off before any analysis is undertaken. The analysis will focus on descriptive statistics and confidence interval estimation. All analyses will be conducted on the intention-to-treat population, in which all participants will be included in the analysis according to the randomised allocation of the service they were recruited from, and regardless of non-adherence with the intervention or withdrawal from the trial. Final analysis will be conducted when all available outcome data has been received. All summaries will be presented overall, by arm and by cluster (where relevant) using frequencies and summary statistics. The number of participants with missing data will be presented. All participant reported outcome measures will be scored according to relevant scoring manuals and summarised overall, and by arm at each time-point. Point estimates and 95% confidence intervals for the difference in outcomes between arms will be presented. No inferential testing is planned.

The primary outcome for the definitive trial is pain intensity as measured by the BPI; a ≥ 2 point reduction between baseline and 1-week follow-up would be considered a clinically significant change in pain intensity. [34] To generate evidence of proof of principle, the mean change from baseline in the 1-week, 1-month and 2-months BPI scores in the two arms will be reported, together with a range of confidence intervals around the main estimate to inform us as to the likelihood of where the ‘true’ estimate may lie. Analysis will adjust for the minimisation factors.

This trial is not powered to provide a precise estimate of the levels of clustering relating to intervention group effects but it will allow an investigation of this effect [35]. We will estimate the Intra-cluster correlation coefficient (ICC) and produce a range of confidence intervals around this to inform the sample size of the definitive trial.

Pre-defined progression criteria (Table 2) will be used to judge whether it is feasible to progress to a larger definitive trial. While these are the main considerations for the decision to progress to a definitive phase III trial, we will also consider data from all primary and secondary endpoints, and any issues related to successful trial delivery, to determine the feasibility of progressing.

**Table 2:**
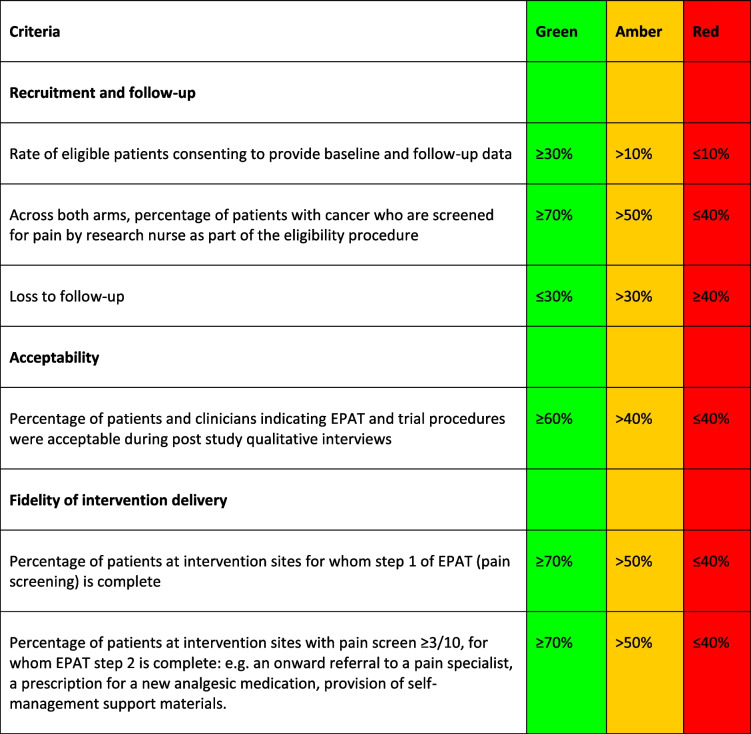
Progression criteria used to judge feasibility of progressing to a definitive trial

^*^Green (go) RCT is feasible with no changes to design or procedures, *Amber *(modify) RCT is feasible following minor enhancement of procedures, *Red *(stop) RCT is not feasible

The economic evaluation will adopt a cost-utility framework, NHS and social care perspective and lifetime horizon (although results from alternative time horizons and wider perspectives will be presented). Discounting will be applied for outcomes beyond 12 months (at 3.5% p.a). We will present incremental cost-effectiveness ratios (ICERs) and net benefit for EPAT plus usual care vs. usual care and explore uncertainty through deterministic and probabilistic sensitivity analyses. Results will be presented as cost-effectiveness planes and acceptability curves [36]. We will use the value of information framework to estimate the value of further research investment (i.e. future trials) [37].

### Process evaluation

The process evaluation will assess the fidelity and quality of trial processes and intervention implementation. It will identify contextual factors associated with variation in intervention uptake, outcome measures and trial processes. The process evaluation will use the qualitative data from the end-of-trial interviews combined with quantitative summaries of recruitment and follow-up data.

Semi-structured interviews with participants, intervention champions and healthcare professionals from both arms will be conducted to investigate the acceptability and fidelity of the intervention components and trial processes (i.e., screening, recruitment, data collection procedures at sites). Issues related to the uptake, use and acceptability of EPAT (including barriers and facilitators to use), as well as adherence and changes in clinicians’ pain assessment practice or participants’ pain control (e.g. access to self-management resources, tailored analgesic prescribing) and fidelity of delivery and contamination will be explored. Participant experiences of completing trial questionnaires will be assessed to inform the feasibility of collecting such data for a larger trial. Interviews schedules will be guided by the framework of acceptability [38]. Interviews lasting up to 1-h will be conducted via telephone, video calling software or in-person. Interviews will be audio-recorded, transcribed, and analysed using Braun & Clarke’s Thematic Analysis [39]. Regular meetings will be held between the research team, co-applicants and Patient and Public Involvement and Engagement (PPIE) group to discuss the developed themes and resolve discrepancies during coding. Any discrepancies in coding will be resolved by the two researchers and if this is not possible, by the wider research team.

### Patient and public involvement and engagement

The PPIE group comprises of five patients with experience of living with and managing chronic cancer pain at home. The PPIE group contributed to the design and planned delivery methods of the trial including adaptation of the intervention components and provided feedback on trial documents and processes.

### Trial organisation and monitoring

The trial is sponsored by the University of Leeds. The day-to-day management of the trial will be overseen by the Trial Management Group (TMG), in line with the standard operating procedures of the CTRU. The TMG comprises of the CI (MM), RF (OR), the CTRU team and other key external members involved in the trial. The TMG will meet monthly. The Trial Steering Committee (TSC), with an independent Chair, will provide overall supervision of the trial and will monitor trial progress, adherence to protocol, participant safety and consideration of new information. The TSC will also include at least two other independent members and a consumer representative. The TSC will meet annually as a minimum and will be provided with reports prepared by the CTRU according to an agreed TSC charter. The CI and other members of the TMG may attend the TSC meetings and report on progress. For a trial of this nature, a separate Data monitoring and Ethics Committee is not required as the TSC will adopt a safety monitoring role.

### Dissemination

Results from the trial will be disseminated through oral and poster presentations at conferences in addition to publications in peer review journals and other forms of media.

We will work with our PPIE group to develop dissemination materials that can be shared with participants that took part in the trial and the wider public. Results will be disseminated to all participating outpatient services and to the trial funder via face-to-face meetings and/or electronic methods.

### Data management

All data collection forms that are transferred to or from the CTRU will be coded with a trial number and two participant identifiers, the participants’ initials, and date of birth. All information collected during the trial will be kept strictly confidential on paper and electronically at the CTRU and Leeds Institute of Health Sciences (LIHS). Relevant standard operating procedures, guidelines, and work instructions in relation to data management, processing and analysis of data will be followed. If a participant withdraws consent from further collection of data, their data already collected will remain on file and will be included in the final study analysis.

All data will be held by CTRU and LIHS and at the end of the trial this will be securely archived at the University of Leeds in line with the Sponsor’s procedures for a minimum of 5-years. NHS Sites are responsible for archiving all trial data and documents until authorisation is issued from the Sponsor for confidential destruction.

### Ethical review and trial registration

The received research ethical approval from South Yorkshire Research Ethics Committee and Health Research Authority (21/HRA/5245) and site-specific approval will be requested from the appropriate research and innovation offices at each site. The trial will be conducted in accordance with the principles of Good Clinical Practice (GCP) in clinical trials, as applicable under UK regulations, the UK Policy Framework for Health, and Social Care Research, and through adherence to Sponsor and CTRU Standard Operating Procedures (SOPs), as defined in the Delegation of Responsibilities. Protocol amendment will be communicated to all participating sites via email from the project team. The trial is registered on the ISRCTN registry (86,926,298).

## Discussion

The CAPTURE pilot trial opened to recruitment in December 2023 and recruitment is ongoing. The trial will evaluate the feasibility of conducting a phase III cluster randomised controlled trial of a standardised pain management programme (EPAT) integrated within routine care at NHS oncology outpatient services.

Previous evidence from clinical trials shows that standardising pain assessment in oncology leads to improvements in cancer patients’ pain and quality of life [10]. However, there is little literature describing the optimal ways to implement standardised pain assessment within routine care pathways in oncology outpatient services [40]. This pilot trial will address this research gap.

The strengths of the pilot trial relate to the sample size and number of clusters that will be recruited. Involving multiple sites (i.e. hospitals) and clusters (i.e. outpatient services) will help to optimise allocation of future resources by identifying and addressing logistical challenges and refining the intervention before conducting a larger-scale trial. The pilot trial will also determine the feasibility of future economic evaluation of EPAT and provide preliminary cost-effectiveness estimates. Including an embedded process evaluation enables in-depth exploration of intervention implementation, mechanisms of impact and fidelity from a participant, healthcare professional and researcher perspective. Conducting this multi-centre, cluster randomised pilot trial will provide a robust exploration of the adapted pain assessment intervention (EPAT) and an understanding of how it can be implemented in a complex healthcare system.

We acknowledge that a limitation of the trial design is that findings will be specific to the United Kingdom National Health Service. It may therefore be difficult to generalise results to other healthcare systems worldwide. There may be potential for selection bias as participants are recruited post cluster randomisation. There is also potential for contamination between clusters if healthcare professionals rotate between services and share information about the intervention. These issues will be considered carefully during the trial and will be explored within the process evaluation.

## Conclusions

This multi-centre, cluster randomised pilot trial will provide useful information to aid the design of a future definitive phase III trial to evaluate the clinical and cost-effectiveness of a standardised pain assessment tool in oncology outpatient services within the UK National Health Service.

## Data Availability

Data access requests should be made to CTRU-DataAccess@leeds.ac.uk.
